# The Conflict within and the Escalating War between the Sex Chromosomes

**DOI:** 10.1371/journal.pgen.1002955

**Published:** 2012-09-13

**Authors:** Jeffrey M. Good

**Affiliations:** Division of Biological Sciences, University of Montana, Missoula, Montana, United States of America; University of Arizona, United States of America

Selfish genetic elements that distort Mendelian segregation to favor their own transmission are common in eukaryotic genomes [1], [2]. Segregation distortion can reduce whole organism fitness, resulting in strong counter selection for genes that suppress distorters. Such intragenomic conflicts have the potential to drive recurrent bouts of antagonistic co-evolution [3]. Theory predicts that genetic conflicts should be particularly intense between the sex chromosomes [4], [5]. The expectation that sex-linked conflict should be rampant has led to a renewed emphasis on the importance of antagonistic co-evolution for driving genome evolution [6]. However, while numerous examples of genes involved in intragenomic conflict now exist [1], evidence for antagonistic co-evolution between the mammalian X and Y chromosomes has remained elusive.

In this issue of *PLOS Genetics*, Cocquet et al. have demonstrated a genetic basis for X–Y conflict acting during a crucial stage of mouse spermatogenesis [7]. The sex chromosomes are silenced via chromatin remodeling during the initiation of meiosis (meiotic sex chromosome inactivation or MSCI) [8]. Gene silencing persists through the remainder of spermatogenesis (postmeiotic sex chromatin or PMSC), save for a subset of genes that escape inactivation [9].

Considerable progress has been made recently on the epigenetic regulation of MSCI and PMSC, including the identification of a multicopy Y-linked gene, *Sly*, involved in the maintenance of PMSC [10]. Male mice with *Sly* deficiency show up-regulation of several sex-linked genes during PMSC, are sub-fertile, and produce female-biased litters. Thus, *Sly* is a strong candidate for being involved in X–Y conflict due to its repressive interaction with other genes and its potential to bias sex chromosome transmission. Intriguingly, there are two X-linked genes (*Slx* and *Slxl1*; hereafter *Slx*/*Slxl1*) that are closely related to *Sly*. Both are regulated by *Sly*, occur in large multicopy clusters on the X, and are crucial for spermatogenesis [11].

**Figure 1:**
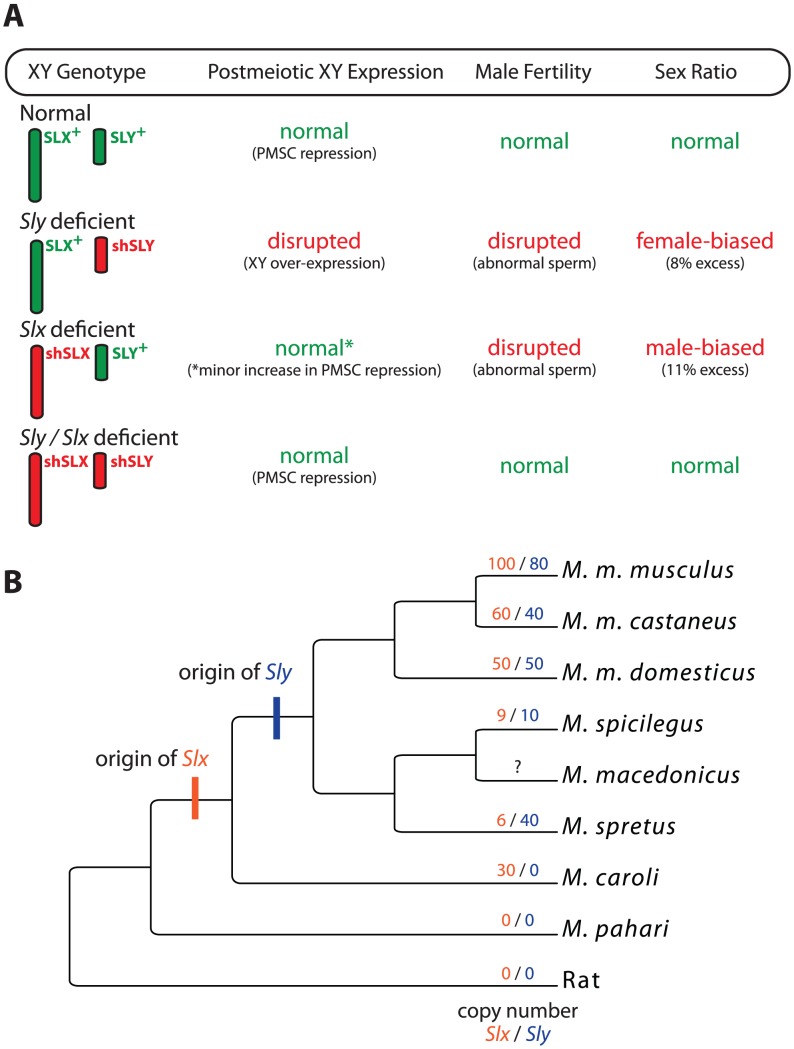
The interaction and evolution of *Sly* and *Slx*/*Slxl1*. (A) A summary of the results from the various deficiency models generated by Cocquet et al. [7]. X and Y chromosome genotypes are given along the margin with wild-type genotypes in green and deficiency genotypes in red (shSLX and shSLY). Two transgenic constructs were made to target *Slx*/*Slxl1* but are presented together here for clarity. For each genotype, the general status of XY expression, male fertility, and sex ratio are given. Phenotypes falling severely outside the wild-type range are in red. (B) Evolutionary relationships among some of the mouse species in the genus *Mus*, following [19]. The branches are not to scale and not all species of *Mus* are shown. Most standard laboratory strains, including those used by Cocquet et al. [7], are derived from *M. m. domesticus*. Inferred copy numbers for *Slx* (orange) and *Sly* (blue) [13] are given for each lineage. *Slxl1* is not shown.

To test for genetic conflict between these genes, Cocquet et al. generated transgenic mice expressing short hairpin RNA (shRNA) that knockdown *Sly* or *Slx*/*Slxl1* transcript levels without completely knocking out gene function [7]. Both *Sly*- and *Slx*/*Slxl1*-deficient mice showed impaired spermatogenesis, but *Slx*/*Slxl1* deficiency led to a slight reduction in sex-linked gene expression in postmeiotic cells and male-biased litters (Figure 1A). Strikingly, mice deficient for both *Sly* and *Slx*/*Slxl1I* showed a complete rescue of XY expression, male fertility, and sex ratio phenotypes. That is, the genes have antagonistic roles during spermatogenesis: *Sly* represses XY expression during PMSC and promotes the transmission of the Y, while *Slx*/*Slxl1* activates XY expression and promotes the transmission of the X. The surprising conclusion is that antagonism depends on the relative expression of these genes and not their total abundance.

Several questions remain regarding the mechanistic and genetic bases of distortion. For example, segregation distortion in the *Sly*-*Slx*/*Slxl1* system appears to be caused by the differential fertilization ability of X- and Y-bearing sperm. Distorter genes often skew transmission through epistatic interactions with one or more responder genes [12]. In this context both *Sly* and *Slx*/*Slxl1* appear to be distorters acting on one or more responder genes to impair the function of the X- or Y-bearing sperm, respectively [7]. Which raises the question, what are the responders?

Even more interesting are the evolutionary consequences of recurrent sex-linked conflict. If *Sly* and *Slx*/*Slxl1I* were locked in an antagonistic conflict, then we would predict that each would be rapidly evolving on some level. The relevant metric here seems to be gene copy number. *Sly* and *Slx*/*Slxl1I* are recent additions to the mouse genome, appearing within the past 3 million years (Figure 1B). Since that time they have rapidly expanded in some, but not all, lineages [13]. Why? Is genetic conflict more intense in some species? Or is the antagonistic interaction a consequence of novel functions that have evolved more recently? The *Mus musculus* X is enriched for dozens of other multicopy gene families expressed primarily in postmeiotic cells, which is thought to be a mechanism for escaping PMSC [14]. This interpretation now appears to be correct, with the added caveat that the entire process may be a side effect of genetic conflict between *Sly* and *Slx*/*Slxl1I*. Most of these X-linked amplicons are repressed by *Sly* during PMSC. Thus, the rapid expansion of *Sly*—driven by conflict with *Slx*/*Slxl1I*—may in turn drive compensatory expansion of other sex-linked genes in order to maintain proper expression levels [13].

One important consequence of recurrent sex-linked conflict is its potential to drive speciation [6]. Several of the mice presented in Figure 1B can hybridize, often resulting in hybrid male sterility (HMS). In particular, some reciprocal crosses between *M. m. musculus* and *M. m. domesticus* yield asymmetric HMS; males are only sterile when a *M. m. musculus* female is crossed with an *M. m. domesticus* male. Moreover, sterile males show widespread over-expression of the X, presumably due to a failure of MSCI and/or PMSC [15]. Cocquet et al. [7] propose that interactions between *Sly* and *Slx*/*Slxl1I* may be the cause of this HMS because copy number differences between the subspecies will yield hybrid males that are *Sly* deficient [7]. While this model is intriguing, it must be considered in light of recent work showing that HMS between *M. m. musculus* and *M. m. domesticus* is genetically complex and not strongly dependent on the origin of Y [16], and that other genetic interactions causing HMS also disrupt XY gene expression [17]. Nonetheless, these data do not exclude an important contribution of *Sly*/*Slx* mismatch to HMS in this or any other mouse hybrid crosses. If true, this would provide the first direct evidence that sex-linked genetic conflict can drive mammalian speciation.

Finally, the finding that a few novel genes control epigenetic regulation of a key step in spermatogenesis is quite remarkable. The basic epigenetic processes underlying PMSC appear to be conserved within mammals, yet its genetic regulation has only been elucidated in mice [10]. These insights are exciting, but are tempered by the fact that the key genes regulating PMSC in mice do not exist in the vast majority of mammals. The human X and Y show similar patterns of PMSC repression, including escape from silencing of several single and multicopy genes [18]. However, fewer than 20% of these genes are shared with mouse. Collectively, these findings illustrate the power of evolution to generate novelty in the face of developmental constraint and call into question the notion that research on a few model systems will be sufficient to elucidate the general molecular underpinnings of reproduction. When it comes to the evolution of reproduction and the sex chromosomes, exceptions may prove to be the rule.
